# Advances in endoplasmic reticulum autophagy and its role in viral infection

**DOI:** 10.3389/fimmu.2026.1966606

**Published:** 2026-10-28

**Authors:** Yulin He, Yaxing Su, Qing Xu, Zhibiao Bian, Pinpin Chu, Chunling Li, Shao-lun Zhai, Yan Li, Zhiyong Jiang, Xia Zhou, Hongchao Gou, Huahua Kang

**Affiliations:** 1 Institute of Animal Health, Guangdong Academy of Agricultural Sciences, Guangdong Provincial Key Laboratory of Livestock Disease Prevention, Scientific Observation and Experiment Station of Veterinary Drugs and Diagnostic Techniques of Guangdong Province, Guangdong Provincial Key Laboratory of Research on the Technology of Pig-breeding and Pig-disease prevention, Guangzhou, China; 2 College of Veterinary Medicine, South China Agricultural University, Guangdong Laboratory for Lingnan Modern Agriculture, Guangzhou, China

**Keywords:** ER-phagy, ER-phagy receptors, IFN pathway, innate antiviral immunity, selective autophagy, virus–host interactions

## Abstract

Endoplasmic reticulum autophagy (ER-phagy) is an emerging form of selective autophagy that maintains ER homeostasis by selectively delivering ER components to lysosomes for degradation. Increasing evidence indicates that ER-phagy is closely involved in immune regulation, particularly in the context of viral infection, where it influences host antiviral defenses and virus–host interactions. In this review, we summarize recent advances in ER-phagy research, focusing on the identified mammalian ER-phagy receptors, their molecular characteristics, and their roles in maintaining ER function. Furthermore, we discuss the bidirectional interplay between ER-phagy and viral infections, including how ER-phagy contributes to innate immune responses through the regulation of antiviral signaling pathways, and how viruses manipulate ER-phagy machinery to promote their replication or evade host immunity. By highlighting the immunological significance of ER-phagy in virus–host interactions, this review provides new perspectives on the role of ER quality control mechanisms in antiviral immunity and their potential implications for the development of antiviral strategies.

## 1 Introduction

Autophagy is a highly conserved lysosome-dependent degradation pathway in eukaryotic cells that maintains cellular homeostasis by removing damaged or superfluous proteins, organelles, and macromolecules, thereby recycling their constituents for cellular reuse (1). The phenomenon of autophagy was first described by Porter and Ashford in 1962. Initially, autophagy was considered a non-selective bulk degradation process; however, the subsequent identification of autophagy receptors has led to the recognition of selective autophagy as a distinct and regulated process (2). To date, multiple forms of selective autophagy have been characterized, including mitophagy (3), aggrephagy (4), ribophagy (5), pexophagy (6), lipophagy (7), lysophagy (8), and ferritinophagy (9).

The endoplasmic reticulum (ER) is an essential organelle in eukaryotic cells that plays central roles in protein folding and modification, calcium homeostasis, and lipid biosynthesis (10). Endoplasmic reticulum autophagy (ER-phagy) refers to the selective degradation of portions of the ER via the autophagy–lysosome pathway. The concept of ER-phagy was first introduced by Bernales et al. in 2007 (11), and its mechanistic distinction from canonical autophagy was further emphasized by Schuck in 2014 (12). ER-phagy contributes to the maintenance of ER homeostasis by selectively removing excess, damaged, or dysfunctional ER subdomains, thereby representing an important component of the ER quality-control network. In this context, ER-phagy acts in parallel with, and is complementary to, the ER-associated degradation (ERAD) system, which primarily targets misfolded proteins within the ER lumen (13).

As obligate intracellular parasites, many viruses hijack ER functions to support their replication cycles, including viral protein processing, membrane remodeling, and virion assembly (14, 15). In recent years, accumulating evidence has indicated that ER-phagy is involved in the regulation of viral infection, where it may influence viral replication cycles and host antiviral responses. In this review, we summarize the current understanding of ER-phagy, with a focus on mammalian ER-phagy receptors and the role of ER-phagy in virus–host interactions.

## 2 ER-phagy and ER-associated lysosomal degradation pathways

The endoplasmic reticulum (ER) is the major site in the cell for protein folding and trafficking and is central to many cellular functions (16). Physiological or pathological stimuli lead to ER stress, unfolded or misfolded proteins accumulate within the endoplasmic reticulum (ER) lumen. To clear these aberrant proteins, cells initiate ER quality control (ERQC) systems, which comprise ER-associated degradation (ERAD) and ER-to-lysosome-associated degradation (ERLAD). ERAD primarily recognizes misfolded or aberrant ER proteins and retrotranslocates them from the ER to the cytosol for ubiquitin-dependent proteasomal degradation. In contrast, ERLAD is a broader term describing lysosome-directed degradation from the ER, serving as a supplementary pathway to degrade the ERAD-resistant misfolded proteins through vesicular or autophagic mechanisms (17, 18). ER-phagy refers to the selective degradation of portions of the endoplasmic reticulum (ER) through autophagy- or lysosome-dependent pathways (19). Depending on the route of delivery to lysosomes, ER-phagy can be broadly categorized into macro-ER-phagy and micro-ER-phagy (20).

### 2.1 Macro-ER-phagy and micro-ER-phagy

In macro-ER-phagy, ER fragments are selectively recognized and sequestered into double-membrane autophagosomes, which subsequently fuse with lysosomes for degradation. This pathway is typically receptor-dependent and represents the canonical form of ER-phagy in mammalian systems. In contrast, micro-ER-phagy involves the direct engulfment of ER portions by lysosomes through membrane invagination or direct uptake, which may occur independently of autophagosome formation under certain conditions (20). (**Figure 1**).

**Figure 1:**
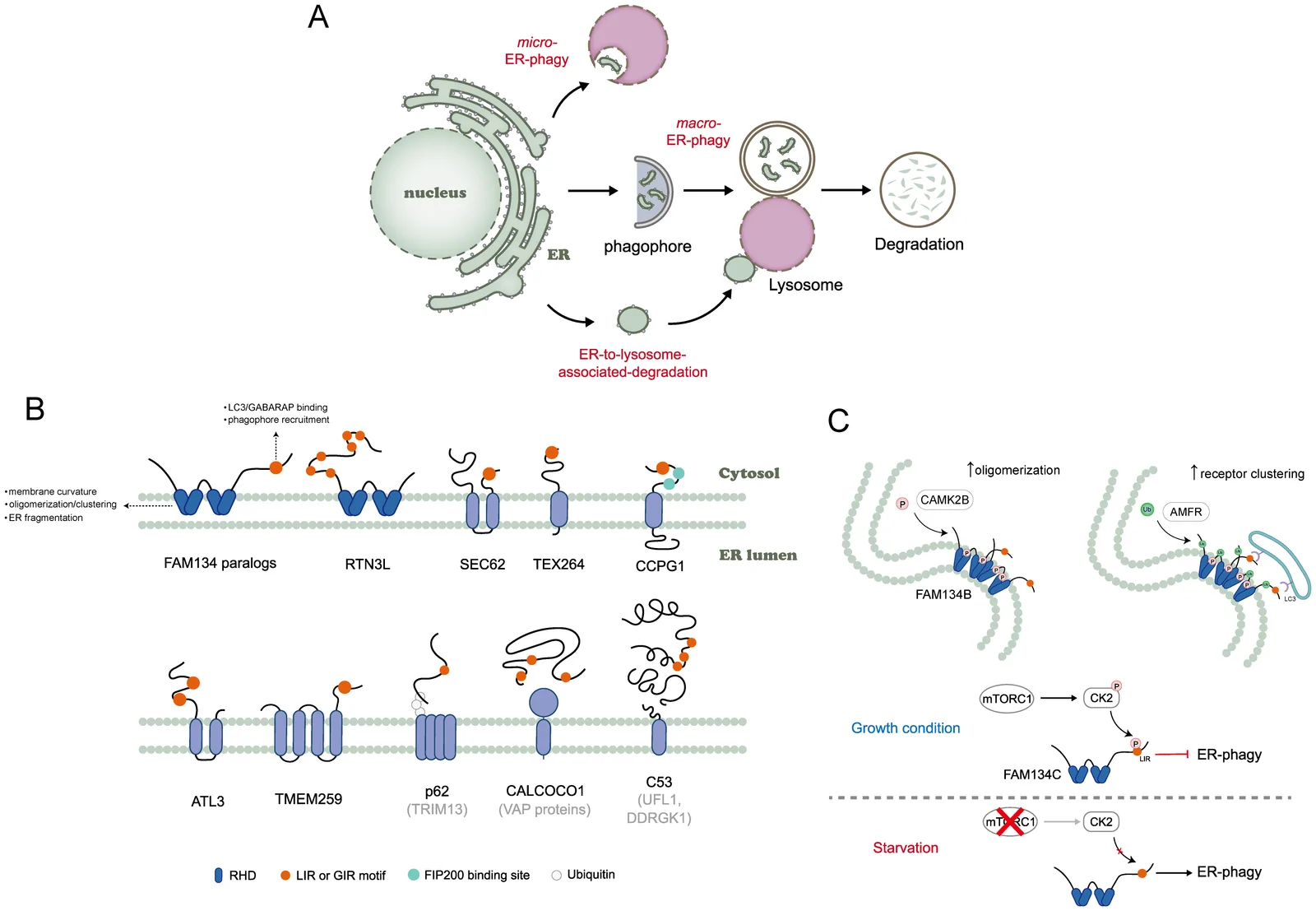
ER quality-control pathways and ER-phagy receptors. **(A)** In macro-ER-phagy, ER fragments are recognized by specific ER-resident receptors, sequestered into a double-membrane autophagosome, and subsequently delivered to the lysosome for degradation. In micro-ER-phagy, portions of the ER are directly engulfed by the lysosomal or vacuolar membrane through invagination or protrusion, internalizing the cargo without forming an intermediate autophagosome. In ERLAD, ER-derived vesicles directly fuse with the lysosome, leading to the degradation of the vesicular contents. **(B)** FAM134 family proteins, RTN3L, SEC62, TEX264, CCPG1, ATL3, and TMEM259 are ER-resident ER-phagy receptors. p62, CALCOCO1, and C53 are soluble cytosolic ER-phagy receptors. TRIM13, VAP proteins, UFL1, and DDRGK1 are ER-phagy-associated regulatory/accessory factors. **(C)** CAMK2B mediated phosphorylation and AMFR-dependent ubiquitination enhance the oligomerization and receptor clustering of FAM134B. FAM134C is regulated by CK2-dependent phosphorylation near its LIR domain: under nutrient-rich conditions, phosphorylation reduces LC3 binding, whereas starvation-induced mTORC1 inhibition decreases CK2-dependent phosphorylation and promotes FAM134C-mediated ER-phagy.

Unless otherwise specified, ER-phagy in this review refers primarily to macro-ER-phagy. This process is characterized by several key features: (i) selective fragmentation and remodeling of ER membranes; (ii) receptor-mediated recognition of ER subdomains; (iii) spatial and functional diversity of ER-phagy receptors across different ER regions; and (iv) dynamic regulation of ER-phagy activity in response to physiological and stress conditions (21). These features collectively ensure the precise control of ER homeostasis and establish the framework for receptor-mediated ER quality control, which will be discussed in the following section.

### 2.2 Non-autophagic ERLAD

In addition to canonical ER-phagy, ERLAD also encompasses a non-autophagic, vesicle-mediated degradation pathway. Although also dependent on specific ER-phagy receptors, this distinct ERLAD pathway does not require the engulfment of ER membranes into double-membrane autophagosomes. Instead, it relies on ER-derived single-membrane vesicles that transport and fuse with the endolysosomes, releasing their luminal content within endolysosomes for degradation (22).

## 3 ER-phagy receptors in mammals

ER-phagy in mammals is mediated by a diverse group of receptors and receptor-associated proteins that enable the selective recognition and delivery of ER subdomains to the lysosomal degradation system. Based on their subcellular localization and membrane topology, mammalian ER-phagy receptors can be broadly classified into ER membrane-resident and soluble/cytosolic proteins. ER membrane-resident receptors include members of the FAM134 family, RTN3L, SEC62, TEX264, CCPG1, ATL3, and TMEM259. Soluble/cytosolic receptors such as CALCOCO1 function in specific ER regions. These receptors couple ER membranes to the autophagic or lysosomal degradation machinery through LC3/GABARAP-interacting motifs or other receptor-specific mechanisms (**Figure 1**). In addition, several accessory or regulatory proteins, such as TRIM13, AMFR, and ARL6IP1, modulate ER-phagy by regulating ubiquitination, receptor clustering, or ER membrane remodeling.

### 3.1 ER membrane resident receptors

#### 3.1.1 FAM134 family (FAM134A/B/C)

The FAM134 family comprises three ER-resident ER-phagy receptors, FAM134A, FAM134B, and FAM134C, which share a reticulon homology domain (RHD) and a cytosolic LC3-interacting region (LIR), but exhibit distinct activation properties and physiological functions (23). Among them, FAM134B/RETREG1 is the best-characterized member and plays a central role in ER membrane remodeling during ER-phagy.

The RHD of FAM134B contains two transmembrane hairpins and amphipathic helices that promote membrane curvature and remodeling (24). Upon ER-phagy induction, FAM134B undergoes oligomerization and clustering within the ER membrane, which amplifies RHD-dependent membrane curvature and promotes the fragmentation or scission of ER membranes (24, 25). FAM134B oligomerization therefore links receptor activation to physical ER membrane remodeling. In parallel, the cytosolic LIR motif of FAM134B binds LC3/GABARAP proteins on the nascent phagophore, thereby coupling the remodeled ER membrane to the autophagic machinery (26). Consequently, the RHD and LIR domains perform complementary functions: the RHD promotes ER membrane curvature and fragmentation, whereas the LIR recruits the autophagic machinery to facilitate sequestration of the ER fragments. The coordinated action of these domains enables FAM134B to function as both an ER membrane-remodeling factor and an ER-phagy receptor. In addition, ARL6IP1 has been reported to interact with FAM134B to form heteromeric clusters, a process required for efficient ER membrane remodeling and the maintenance of normal ER-phagy (27).

FAM134B activity is further regulated by post-translational modifications. Under ER stress, Ca^2+^/calmodulin-dependent protein kinase II beta (CAMK2B) phosphorylates the RHD region of FAM134B, enhancing receptor oligomerization and membrane fragmentation, thereby increasing ER-phagy activity (25). In addition, phosphorylation-dependent ubiquitination of FAM134B promotes the formation of high-density receptor clusters, which facilitates ER membrane remodeling and ER-phagy flux (28, 29). AMFR has been identified as an E3 ubiquitin ligase that contributes to FAM134B ubiquitination within receptor clusters, linking post-translational modification to receptor clustering and ER remodeling (29).

FAM134C exhibits a distinct mode of regulation. Under nutrient-rich conditions, casein kinase 2 (CK2)-mediated phosphorylation of residues flanking its LIR motif reduces its affinity for LC3 and restrains FAM134C-mediated ER-phagy. Upon starvation, inhibition of mTORC1 reduces CK2-dependent phosphorylation of FAM134C, thereby increasing its interaction with LC3 and promoting ER-phagy (30). More recent evidence further indicates that CK2-dependent phosphorylation of FAM134C, together with phosphorylation-dependent ubiquitination, regulates the formation of high-density FAM134 receptor clusters and ER-phagy flux (28). Thus, phosphorylation can either activate or restrain FAM134-dependent ER-phagy depending on the receptor, phosphorylation site, and cellular context.

#### 3.1.2 RTN3L

Reticulon 3 long isoform (RTN3L) functions as a selective ER-phagy receptor enriched in tubular ER domains. RTN3L contains multiple LIR motifs in its N-terminal region, enabling efficient interaction with LC3/GABARAP proteins. It promotes ER tubule fragmentation and facilitates selective sequestration of tubular ER into autophagosomes (31). RTN3L activity is also linked to ER stress signaling pathways such as IRE1α activation, which coordinates ER remodeling during proteostasis stress (32). In addition, RTN3L has been implicated in ER–endosome contact site regulation, suggesting a broader role in membrane organization (33).

#### 3.1.3 SEC62

SEC62 is a component of the ER translocon complex involved in protein translocation into the ER (34). It was subsequently identified as an ER-phagy receptor that mediates ER turnover during recovery from ER stress (35). SEC62 contains a conserved LC3-interacting region (LIR) within its C-terminal cytosolic domain and is involved in ER-phagy during recovery from ER stress by selectively delivering ER components to autolysosomes for degradation (35). Furthermore, overexpression of SEC62 restores ER-phagy activity and markedly alleviates Alzheimer’s disease (AD) phenotypes (36).

#### 3.1.4 TEX264

TEX264 is a widely distributed and expressed ER-phagy receptor.TEX264 contains a single transmembrane domain and a LIR motif (37). Phosphorylation of its LIR motif is critical for interaction with ATG8 proteins and recruitment to autophagosomes (38). In addition, TEX264 possesses a long intrinsically disordered region (IDR), which is critical for its receptor function, bridging the gap between the ER and autophagosomal membranes. TEX264 plays a dominant role in starvation-induced ER-phagy and is considered one of the most efficient ER-phagy receptors due to its broad ER distribution and strong LC3/GABARAP binding capacity (37, 39).

#### 3.1.5 CCPG1

CCPG1 is a non-canonical ER-phagy receptor that integrates signals from the unfolded protein response, linking ER stress to ER-phagy. It contains a canonical LIR motif and a FIP200-interacting region; both of these interactions facilitate ER-phagy (40). In addition, CCPG1 possesses several highly conserved cargo-interacting regions (CIRs), enabling selective degradation of ER-resident proteins (41). *In vivo*, CCPG1 prevents the hyper-accumulation of insoluble protein within the ER lumen of pancreatic acinar cells and alleviates ER stress (40, 42).

#### 3.1.6 ATL3

Atlastins are ER-resident GTPases, including ATL1, ATL2, and ATL3, that mediate ER membrane fusion and play a dual role in ER morphology and ER-phagy (43–45). Among them, ATL3 has been identified as an ER-phagy receptor interacting with GABARAP but not LC3 through two GABARAP-interacting motifs (GIMs), promoting tubular ER degradation upon starvation (46). ATL proteins cooperate with other receptors such as FAM134B to facilitate ER fragmentation and segregation (45). ATL2 and ATL3 also interact with the ULK1 complex, suggesting a role in early autophagosome formation and ER remodeling coordination (47).

#### 3.1.7 TMEM259

TMEM259 (Membralin) is an ER-resident multi-pass membrane protein initially characterized as a component of the ERAD network that contributes to ER stress alleviation (48). More recently, TMEM259 has been identified as a non-canonical ER-phagy receptor that mediates ERLAD by targeting the foreign glycoproteins. This activity depends on a functional LC3-interacting region (LIR) motif within its cytoplasmic tail, which mediates autophagic cargo delivery. Mechanistically, TMEM259 assembles a MAN1B1–VCP complex that facilitates the delivery of foreign glycoproteins from the ER to lysosomes for degradation (49).

### 3.2 Soluble/cytosolic receptors

#### 3.2.1 CALCOCO1

CALCOCO1 is a soluble cytosolic ER-phagy receptor that bridges ER-resident proteins and ATG8 family members. It contains LIR- and UDS-interacting region (UIR) motifs that mediate interaction with ATG8 proteins. CALCOCO1 recognizes ER membranes through binding to VAPA/VAPB via a conserved FFAT-like motif, enabling selective degradation of the tubular ER in response to proteotoxic and nutrient stresses (50). Functional studies indicate that CALCOCO1-mediated ER-phagy is regulated by mTOR signaling and contributes to basal ER turnover (51).

#### 3.2.2 p62(SQSTM1)

The cytosolic autophagic receptor p62 is involved in ubiquitin-dependent ER-phagy mediated by the N-degron pathway. In this pathway, the ER-resident transmembrane E3 ubiquitin ligase TRIM13 undergoes K63-linked auto-ubiquitination and recruits p62/SQSTM1. In parallel, ER-resident proteins can undergo N-terminal arginylation, generating Nt-Arg degrons that bind the ZZ domain of p62 and promote oligomerization of TRIM13–p62 complexes. These oligomerized complexes are segregated together with ER membranes and subsequently targeted to autophagosomes for lysosomal degradation (52).

#### 3.2.3 CDK5RAP3 (C53/LZAP)

CDK5RAP3 is a non-canonical ER-phagy receptor, which forms a complex with UFL1 and DDRGK1, and this complex anchors CDK5RAP3 to the ER membrane. CDK5RAP3 has been identified as an ATG8-binding protein that contains noncanonical, shuffled ATG8-interacting motifs (sAIMs) within its IDR. These sAIMs are essential for ATG8 recognition and the initiation of autophagy (53). As a key component of the UFMylation machinery, CDK5RAP3 mediates ER-phagy that is regulated by ubiquitin-fold modifier (UFM1) 1. Mechanistically, upon ribosome collision or stalling at the ER, the interaction between CDK5RAP3 and UFM1 is disrupted, and this disruption enhances the binding affinity of CDK5RAP3 for ATG8. Subsequently, UFM1 is transferred from CDK5RAP3 to ribosomes, thereby activating the C53 pathway to clear toxic polypeptides (53, 54). This molecular switch links the ribosome-associated quality control with selective autophagy at the ER.

## 4 STING-mediated ER-phagy and innate immune signaling

STING is primarily a central adaptor in the cGAS–STING innate immune pathway and can also engage selective autophagy following STING activation (55). It contains LIRs that enable direct binding to LC3 and mediate ER-phagy, for which STING dimerization and cGAMP binding are indispensable (56). In addition, SEC62 acts as an ER-phagy receptor in STING-mediated ER-phagy. Upon STING activation, the interaction between SEC62 and STING is enhanced, whereas ATP2A2 regulates this process by modulating STING polymerization and trafficking (57). These findings highlight a functional link between ER-phagy and innate immune signaling, in which ER-phagy can regulate the abundance and activity of immune signaling components at the ER.

## 5 ER-phagy in virus–host interactions`

The effects of ER-phagy on viral infection are context-dependent and appear to be largely determined by the identity and function of the cargo being targeted. When ER-phagy selectively removes viral proteins or virus-damaged ER structures required for viral replication, it can contribute to antiviral defense and restrict viral propagation. In contrast, ER-phagy or ER-associated lysosomal degradation may promote viral fitness when it targets host restriction factors or components of antiviral signaling pathways (**Table 1**).

**Table 1:** Summary of ER-phagy and ERLAD pathways in viral infections.

Virus	Host model	ER-phagy factor/Pathway	Target Cargo	Infection Stage Affected	Virological/Immunological outcome
EBOV	Mammalian (Human cells)	FAM134B	Viral NP/Nucleocapsids	Replication/Assembly	Antiviral: Restricts viral replication and virion production
EBOV	Mammalian (Human cells)	RNF185-p62/FAM134B	Misfolded/Excess EBOV-GP	viral protein synthesis/assembly	Proviral: Prevents GP cytotoxicity, maintains viral fitness
positive-strand RNA viruses (e.g. coronaviruses)	Mammalian (Human cells)	Sec62	VROs	Genome replication	Antiviral: Clears VROs and restricts viral replication
positive-strand RNA viruses (e.g. BBSV)	Plant (*N. benthamiana*)	NbSec62	Viral p23/VROs	Genome replication	Antiviral: Clears VROs and restricts viral replication
Flaviviruses (DENV/ZIKV)	Mammalian (Human cells)	FAM134B (cleaved by NS2B3)	VROs	Genome replication	Proviral: FAM134B cleavage Inhibits ER-phagy to promote viral replication
MLV	Mammalian (Murine/Human cells)	FAM134B	SERINC5 (Host restriction factor)	Assembly/Envelope incorporation	Proviral: Degrades SERINC5 to increase viral infectivity
PRRSV	Mammalian (Porcine cells)	FAM134B (early)/Nsp5-FAM134B (late)	Viral RNA (early)/RLR factors (late)	Early sensing/Late immune evasion	Dual: Enhances IFN early; attenuates IFN late
SARS-CoV-2/EBOV/IAV/HIV-1	Mammalian (Human cells)	TMEM259 (ERLAD)	Viral Class I fusion glycoproteins	viral protein synthesis	Antiviral: Degrades glycoproteins and blocks infection

### 5.1 ER-phagy restrict viral replication

ER-phagy is increasingly recognized as an important component of intrinsic antiviral immunity, which serves as a host defense mechanism that limits viral replication by selectively degrading virus-induced ER remodeling structures and associated protein complexes. In the mammalian cell model, the ER-resident receptor SEC62 is activated during ER stress and mediates the clearance of excess ER and viral replication complexes via ER-phagy, thereby limiting the replication of positive-strand RNA viruses (58). Similarly, in a plant virus model, infection with positive-strand RNA viruses such as beet black scorch virus (BBSV) induces ER-phagy through the interaction between the viral auxiliary replication protein p23 and the ER-phagy receptor NbSec62, which contributes to the clearance of ER regions containing viral replication organelles (VROs) and restriction of viral replication (59). In addition, FAM134B-mediated ER-phagy restricts Ebola virus (EBOV) replication, as loss of FAM134B resulted in increased production of infectious EBOV and accumulation of viral proteins and nucleocapsid structures (60). These findings support a role for ER-phagy as a host mechanism that counteracts the expansion or remodeling of ER membranes exploited by viruses for replication.

TMEM259/Membralin represents a distinct ER quality-control pathway with antiviral activity. TMEM259 functions as a non-canonical ER-phagy receptor and forms a MAN1B1–VCP complex that selectively targets viral class I fusion glycoproteins that are essential for the viral entry, including SARS-CoV-2 spike, EBOV GP, influenza A virus HA, and HIV-1 Env, for lysosomal degradation. Overexpression of TMEM259 potently suppressed pseudoviral infection (49). This pathway operates through an ER-to-lysosome-associated degradation (ERLAD) mechanism and is mechanistically distinct from canonical FAM134B-dependent macro-ER-phagy (**Figure 2**).

**Figure 2:**
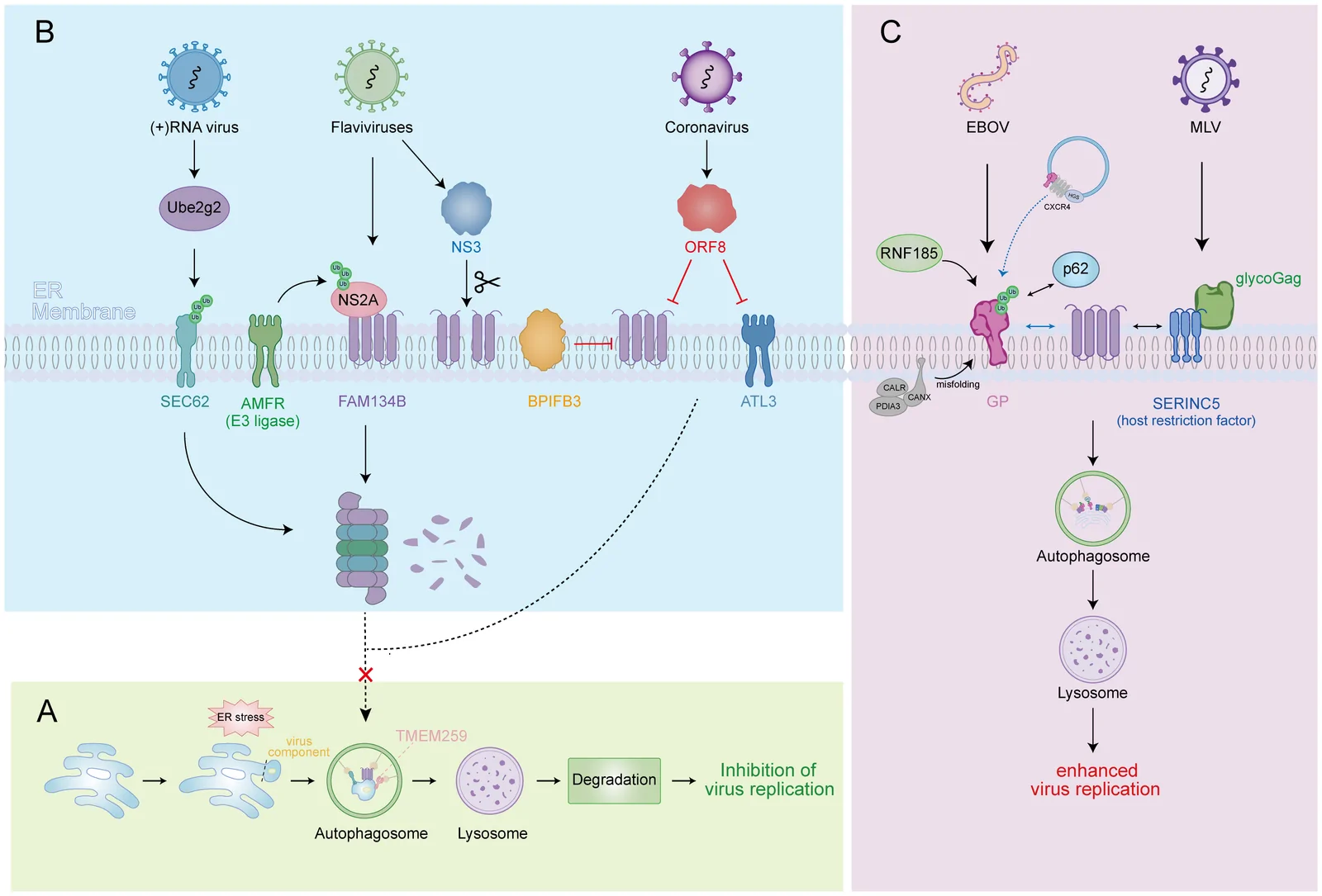
Interactions between ER-phagy and viral infection. **(A)** ER-phagy degrades viral components to restrict viral replication. **(B)** Viruses counteract ER-phagy through diverse mechanisms to facilitate their replication. **(C)** Viruses exploit ER-phagy to maintain viral fitness or enhance viral infectivity.

### 5.2 Viral strategies to antagonize ER-phagy receptors

In turn, viruses have evolved multiple strategies to suppress or evade ER-phagy, thereby preserving ER membranes and protein homeostasis required for viral replication. Positive-strand RNA viruses can inhibit ER-phagy by promoting ubiquitination and degradation of ER stress–related components via the host ubiquitin conjugating protein Ube2g2, including SEC62 and other ER quality control factors, thereby facilitating the formation of viral replication organelles (58).

Flaviviruses employ multiple mechanisms to suppress FAM134B-mediated ER-phagy. The virally-encoded NS2B3 protease complex cleaves FAM134B, preventing ER-phagosome formation (61). In addition, flaviviruses hijack the E3 ligase AMFR to promote ubiquitination and degradation of FAM134B-associated complexes, thereby enhancing viral replication (62). Host factors such as BPIFB3 further restrict FAM134B activity, indirectly promoting flaviviral infection (63).

Coronaviruses have evolved another strategy to interfere with ER-phagy. The viral protein ORF8 suppresses ER-phagy by binding to FAM134B and ATL3 and recruiting these receptors into ORF8/p62 liquid droplets, thereby promoting viral double-membrane vesicle (DMV) formation and replication (64). These findings illustrate that viruses can antagonize ER-phagy not only by directly degrading ER-phagy receptors but also by spatially sequestering them away from the autophagic machinery.

### 5.3 Viral exploitation of ER-phagy pathways

Although ER-phagy generally contributes to ER quality control and antiviral defense, some viruses exploit ER-phagy or related lysosome-directed degradation pathways to eliminate viral proteins or host restriction factors, thereby optimizing their replication and fitness.

During Ebolavirus (EBOV) infection, although the glycoprotein (GP) is essential for viral entry, excessive expression of GP triggers severe cytotoxicity, including cell rounding, detachment, and the downregulation of surface molecules (65–67). To prevent premature cell death and maintain viral fitness, EBOV exploits ER-phagy or the autophagy–lysosomal pathway to downregulate surplus GP levels. Mechanistically, when GP accumulates in the endoplasmic reticulum (ER), PDIA3/ERp57 targets its cysteine residues to induce GP misfolding, thereby triggering the unfolded protein response (UPR). Although misfolded EBOV-GP is recognized by the ER-associated degradation (ERAD) machinery, it is directed to lysosomal rather than proteasomal degradation (68). Specifically, the E3 ubiquitin ligase RNF185 catalyzes K27-linked polyubiquitination of aberrant GP at K673 in its cytoplasmic tail. Polyubiquitinated GP is then recruited to the phagophore assembly site via FAM134B and packaged into autophagosomes via p97 ATPases and p62/SQSTM1, leading to autolysosomal degradation (69). This RNF185–p62 axis represents a crosstalk between ERAD ubiquitination and autophagy–lysosomal pathways. Furthermore, mature GP anchored on the plasma membrane can be retrogradely transported back to the ER. This process is driven by interactions with HGS that hijack the CXCR4 sorting pathway; upon returning to the ER, GP undergoes RNF185-mediated K27- and K63-linked polyubiquitination and is ultimately degraded via FAM134B-dependent ER-phagy (70).

Murine leukemia virus (MLV) provides another example of viral exploitation of ER-phagy. The viral accessory protein glycosylated Gag (glycoGag) binds the host restriction factor SERINC5 in the ER and recruits the ER-phagy receptor FAM134B. FAM134B -dependent micro-ER-phagy selectively eliminates ER-retained SERINC5, thereby reducing the availability of this antiviral restriction factor and enhancing viral infectivity (71).

### 5.4 ER-phagy in innate immune signaling and interferon regulation

Beyond directly affecting viral replication, ER-phagy also modulates innate immune signaling, particularly type I interferon (IFN-I) responses, which indirectly influence the process of infection. The role of FAM134B-mediated ER-phagy in PRRSV infection is context-dependent. In the early stage of infection, physiological ER-phagy promotes viral RNA recognition via TLR3 and RIG-I to trigger type I interferon signaling, ultimately restricting PRRSV replication (72). However, during later stages of infection, PRRSV counteracts this by deploying its non-structural protein 5 (Nsp5) to hijack FAM134B. Instead of suppressing ER-phagy, Nsp5 redirects FAM134B-dependent ER-phagy to selectively degrade key signaling molecules of the RLR pathway, thereby evading host innate immunity (73).

STING serves as a central hub in DNA virus sensing pathways and is subject to regulation by ER-phagy. TEX264-mediated ER-phagy has been shown to promote STING degradation, thereby attenuating IFN-I signaling during DNA viral infection (74). In addition, the G protein-coupled receptor CD97 can recruit FAM134B to initiate ER-phagy-dependent STING turnover. Both mechanisms can suppress antiviral interferon responses and promote virus replication (75) (**Figure 3**).

**Figure 3:**
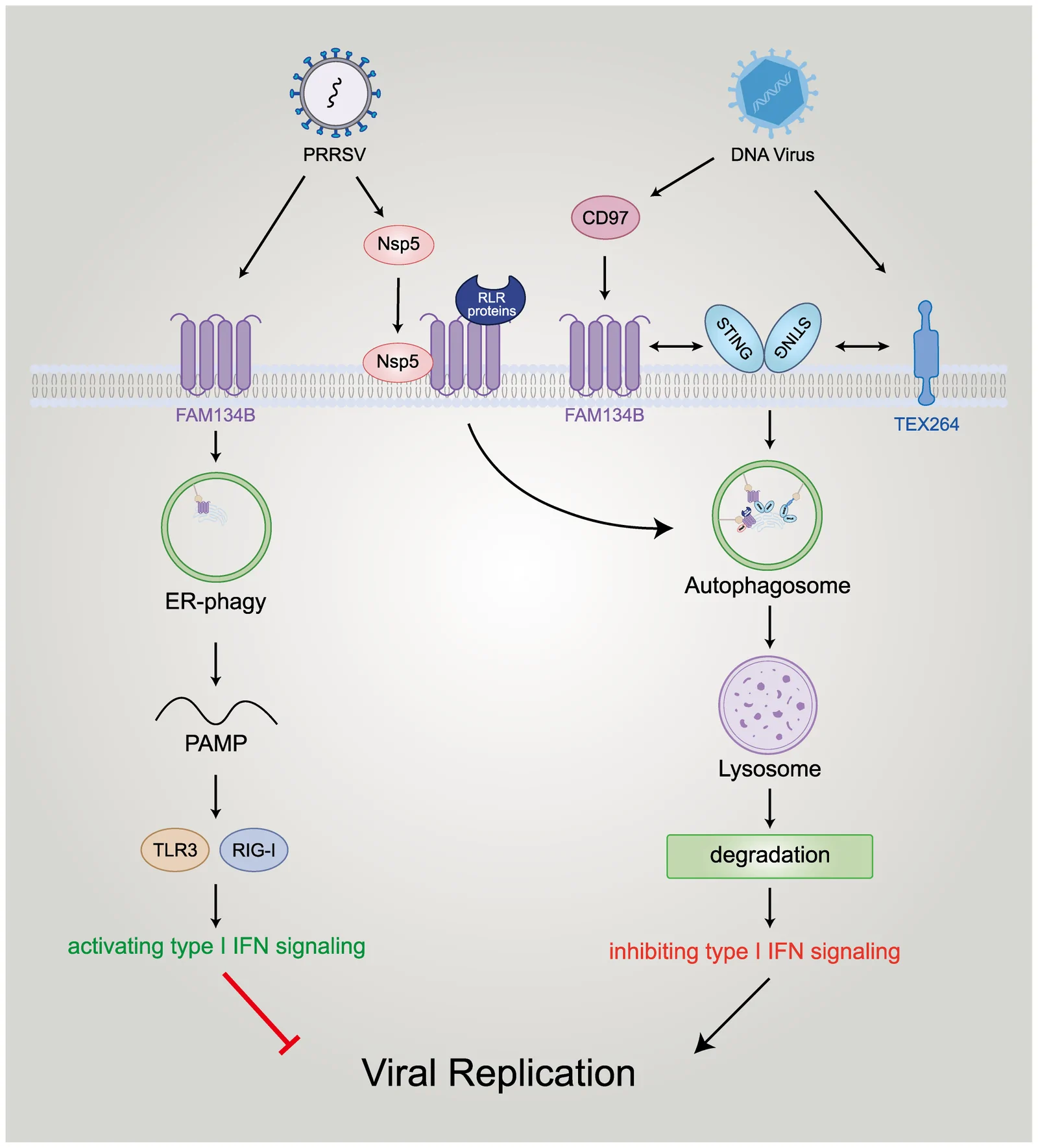
Context-dependent regulation of the interferon (IFN) pathway by ER-phagy during viral infection. ER-phagy exerts dual effects on antiviral immunity: it can enhance IFN signaling to restrict viral replication or promote STING degradation to attenuate IFN responses, depending on the viral infection context.

## 6 Discussion

Despite remarkable advances in ER-phagy research over the past decade, many fundamental questions remain unanswered. A deeper understanding of these issues will not only advance our knowledge of ER homeostasis but also facilitate the development of novel therapeutic strategies.

One major challenge is the continued characterization of the ER-phagy machinery. Although numerous mammalian ER-phagy receptors have been identified, it is unlikely that the current repertoire is complete. Future studies should focus on identifying novel receptors and elucidating how multiple receptors cooperate to recognize distinct ER subdomains and coordinate selective cargo degradation. In addition, the roles of accessory factors, including membrane-remodeling proteins, molecular chaperones, ubiquitin ligases, and cytoskeletal regulators, remain poorly understood and warrant systematic investigation.

Another important direction is to further elucidate the ER-phagy regulation mechanisms. Although ER-phagy is recognized as an essential component of ER quality control, its physiological functions likely extend beyond maintaining ER size and proteostasis. Increasing evidence suggests extensive crosstalk between ER-phagy and other ER quality control pathways, including ER-associated degradation (ERAD), ER-to-lysosome-associated degradation (ERLAD), and the unfolded protein response (UPR). However, how these pathways are coordinated under different physiological and pathological conditions remains largely unknown. Moreover, the upstream signaling networks that determine receptor activation, cargo selection, and ER remodeling, as well as the contribution of post-translational modifications such as phosphorylation, ubiquitination, UFMylation, and acetylation, require further investigation.

An emerging area of particular interest is the role of ER-phagy in virus–host interactions. Recent studies have demonstrated that ER-phagy receptors function as key molecular interfaces between viruses and host cells. While ER-phagy can restrict viral replication by eliminating virus-induced ER remodeling and promoting antiviral signaling, many viruses have evolved sophisticated mechanisms to suppress or exploit ER-phagy for their own benefit. However, it remains unclear why different viruses preferentially target specific ER-phagy receptors and whether receptor selection is determined by viral tropism, replication strategy, or ER membrane architecture. Furthermore, accumulating evidence suggests that ER-phagy exerts context-dependent effects on innate immunity, particularly through regulation of the STING–type I interferon axis, highlighting the complexity of its immunological functions.

From a translational perspective, ER-phagy represents a promising target for host-directed antiviral therapy. Modulating specific ER-phagy receptors or their upstream regulatory pathways may simultaneously disrupt viral replication and restore antiviral immune responses while minimizing the emergence of drug resistance. Nevertheless, because ER-phagy is essential for normal cellular homeostasis, therapeutic interventions must achieve precise spatial and temporal regulation to avoid undesirable effects. Future advances in structural biology, multi-omics technologies, and *in vivo* disease models will undoubtedly deepen our understanding of ER-phagy and accelerate the development of ER-phagy-based therapeutic strategies.
